# Supplementary material for: Pharmacological Treatment of Acute Psychiatric Symptoms in COVID-19 Patients: A Systematic Review and a Case Series
Source: Int J Environ Res Public Health. 2022 Apr 20;19(9):4978. doi: 10.3390/ijerph19094978 (PMC9099660; doi:10.3390/ijerph19094978)
Supplement: Supplementary file 1 [file ijerph-19-04978-s001.zip › ijerph-1566220-supplementary.pdf]

EMBASE

#6	#4 AND #5	1,073
#5	**'coronavirus disease 2019'**/exp	199,510
#4	#1 OR #2 OR #3	52,915
#3	**'acute psychosis'**/exp	1,993
#2	**'delirium'**/exp	37,125
#1	**'restlessness'**/exp	15,063

PUBMED

#3	Search: **#1 AND #2** Sort by: **Publication Date**<br>("psychomotor agitation"[MeSH Terms] OR "psychomotor agitation"[Title/Abstract] OR "delirium"[MeSH Terms] OR "delirium"[Title/Abstract] OR "acute psychosis"[Title/Abstract]) AND ("COVID-19"[MeSH Terms] OR "COVID-19"[Title/Abstract])<br>**Translations**<br>**psychomotor agitation[MeSH Terms]:** "psychomotor agitation"[MeSH Terms]<br>**delirium[MeSH Terms]:** "delirium"[MeSH Terms]<br>**COVID-19[MeSH Terms]:** "covid-19"[MeSH Terms]	523
#2	Search: **(COVID-19[MeSH Terms]) OR (COVID-19[Title/Abstract])** Sort by: **Publication Date**<br>"COVID-19"[MeSH Terms] OR "COVID-19"[Title/Abstract]<br>**Translations**<br>**COVID-19[MeSH Terms]:** "covid-19"[MeSH Terms]	230,938
#1	Search: **((((psychomotor agitation[MeSH Terms]) OR (Psychomotor Agitation[Title/Abstract])) OR (delirium[MeSH Terms])) OR (delirium[Title/Abstract])) OR (Acute Psychosis[Title/Abstract])** Sort by: **Publication Date**<br>"psychomotor agitation"[MeSH Terms] OR "psychomotor agitation"[Title/Abstract] OR "delirium"[MeSH Terms] OR "delirium"[Title/Abstract] OR "acute psychosis"[Title/Abstract]<br>**Translations**<br>**psychomotor agitation[MeSH Terms]:** "psychomotor agitation"[MeSH Terms]<br>**delirium[MeSH Terms]:** "delirium"[MeSH Terms]	28,137

WEB OF SCIENCE

(“Psychomotor Agitation” OR “Delirium” OR “Acute Psychosis”) AND (“COVID-19”)	467
